# An Overview on Target-Based Drug Design against Kinetoplastid Protozoan Infections: Human African Trypanosomiasis, Chagas Disease and Leishmaniases

**DOI:** 10.3390/molecules26154629

**Published:** 2021-07-30

**Authors:** Violeta Kourbeli, Eleni Chontzopoulou, Kalliopi Moschovou, Dimitrios Pavlos, Thomas Mavromoustakos, Ioannis P. Papanastasiou

**Affiliations:** 1 Department of Pharmacy, Division of Pharmaceutical Chemistry, School of Health Sciences, National and Kapodistrian University of Athens, Panepistimioupoli-Zografou, 157 84 Athens, Greece; violetakourbeli@gmail.com; 2 Department of Organic Chemistry, Faculty of Chemistry, National and Kapodistrian University of Athens, Panepistimioupoli-Zografou, 157 71 Athens, Greece; elenichontzo@chem.uoa.gr (E.C.); kmoschovou@chem.uoa.gr (K.M.); dpavlos@chem.uoa.gr (D.P.); tmavrom@chem.uoa.gr (T.M.)

**Keywords:** kinetoplastid protozoan diseases, Human African Trypanosomiasis, Chagas disease, leishmaniases, target-based drug design

## Abstract

The protozoan diseases Human African Trypanosomiasis (HAT), Chagas disease (CD), and leishmaniases span worldwide and therefore their impact is a universal concern. The present regimen against kinetoplastid protozoan infections is poor and insufficient. Target-based design expands the horizon of drug design and development and offers novel chemical entities and potential drug candidates to the therapeutic arsenal against the aforementioned neglected diseases. In this review, we report the most promising targets of the main kinetoplastid parasites, as well as their corresponding inhibitors. This overview is part of the Special Issue, entitled “Advances of Medicinal Chemistry against Kinetoplastid Protozoa (*Trypanosoma brucei, Trypanosoma cruzi* and *Leishmania* spp.) Infections: Drug Design, Synthesis and Pharmacology”.

## 1. Introduction

The protozoan diseases Human African Trypanosomiasis (HAT), Chagas disease (CD), and leishmaniases are infectious diseases, which belong to Neglected Tropical Diseases (NTDs), according to World Health Organization (WHO). These kinetoplastid protozoan infections not only affect low- and middle-income countries (LMICs) like other NTDs, but they span worldwide, because of globalization caused by human and capital translocation. More than 20% of the world’s population lives in an area where NTDs are endemic and over 1 billion people are affected by these diseases each year [1]. Thus, vector and host transportation and new outbreaks happen all around the world. In the last 70 years, public health policy has focused on the eradication of infectious microbes using powerful medical weaponry. Antibiotics and vaccines provoked a bold confidence that the end of infectious diseases was ahead. The devastating COVID-19 pandemic has proven that this grandiose optimism was premature. The evolutionary history of pathogens and human infectious diseases was unpredictably accelerated by the human transformation of global ecosystems and the construction of massive transportation networks connecting human societies worldwide. Emerging infectious diseases are a new threat against human dominance, which might prove more fragile than ever before in history [2].

The kinetoplastid diseases, sleeping sickness (HAT), Chagas disease (CD), and leishmaniases, as well as all NTDs, suffer from impoverished funding and insufficient alignment among relevant partners to achieve overall goals, based on a strategic plan [3]. The treatment of these diseases and overall reduction in burden is a multidisciplinary endeavor that involves changes to health management policies, coordinated public health interventions, and the improvement of health disparities. In 2012, WHO released the first bold roadmap towards 2020, against NTDs. However, glaring gaps remain from these achievements because of failing approaches [4]. A second 2021–2030 road map for NTDs, guided again by WHO, provides an accelerated and ambitious coordination between public organizations and stakeholders towards the prevention, control, elimination, and eradication of these infections [5]. A community of partners, known as Uniting to Combat NTDs, has endorsed and supported the vision of both WHO roadmaps and has updated the London Declaration on Neglected Tropical Diseases [6]. Public–private consortia are forms of cooperation between public authorities and private businesses that carry out funding streams covering the full range of activities required for drug discovery against NTDs [7]. The Drugs for Neglected Disease *Initiative* (DND*i*) is the most successful model of the product development partnership (PDP), which is implicated in drug innovation involving research institutions, pharmaceutical companies, government agencies and international organizations [8,9]. An open-source model example is the Tres Cantos Open Lab Foundation (TCOLF), which allows academics to use GlaxoSmithKline’s R&D facilities in Spain. This GSK-TCOLF partnership has fostered a lot of scientific exchanges, research grants, and follow-up engagement [10]. The Novartis Institute for Tropical Disease (NITD), in cooperation with the Singapore Economic Development Board, have also been involved in a multidisciplinary fight against NTDs [11]. The Wellcome Trust, the Bill and Melinda Gates Foundation, and the NIH also contribute to the research and development against NTDs [12].

The current drugs used against NTDs are suboptimal. The available regimen for the treatment of kinetoplastid diseases is far from satisfactory, with many drugs currently in use dating from early in the 20th century, being essentially poisons, for example, arsenic used to treat sleeping sickness and antimonials for leishmaniasis. The approved therapeutical arsenal presents substantial restrictions, serious adverse side effects, and excessive toxicity, as well as limited efficacy and increasing resistance [13]. Most of the currently used drugs probably have multiple mechanisms of action, as they address to various parasite targets. There is a need for the drug discovery of completely new classes of therapeutic agents, with reduced host toxicity and improved administration profile [14]. The challenge of a typical small-molecule drug discovery follows three pathways [15]:

1. Large-scale cell-based (phenotypic) screening approach. The phenotypic screening identifies drug candidates that are active against the whole cell, without any knowledge of the mechanism of action or a specific cellular or molecular target.
2. Target-based screening approach. There are a few validated targets against the parasitic diseases because of insufficient understanding of the detailed biology of the pathogens.
3. Compound repurposing. Marketed drugs are being tested for redirecting or reprofiling, which has become a recent strategy in medicinal chemistry and drug discovery [16].

In this review, we highlight the challenges associated with target-based drug discovery for the kinetoplastid diseases, Human African Trypanosomiasis (HAT), Chagas disease (CD), and leishmaniases.

## 2. Kinetoplastid Parasites and Related Diseases

### 2.1. The Life Cycles of Trypanosomids

**Figure 1:**
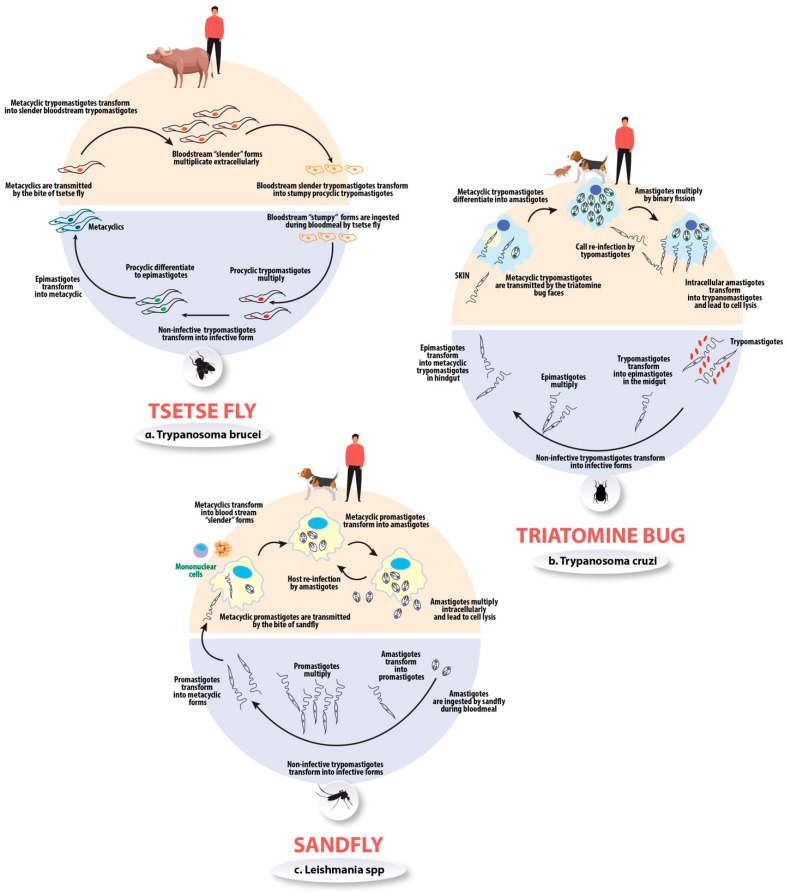
The life cycle of *T. brucei*, *T. cruzi*, and *Leishmania* spp. (**a**) *T. brucei* is transmitted by tsetse flies to humans or livestock animals. The parasites proliferate as procyclic forms, which transform into epimastigotes and then into infectious metacyclic trypomastigotes, in the tsetse fly. These metacyclic trypomastigotes infect mammals, where they differentiate into bloodstream “slender” forms that circulate in the bloodstream, skin, adipose tissue or the brain. The slender forms differentiate into bloodstream “stumpy” (nonreplicative) forms which can be transmitted to the insect vector when it takes a blood meal. (**b**) *T. cruzi*: Initially, an infected triatome insect vector (known as “kissing bug”) takes a blood meal and releases trypomastigotes near the site of bite injury. Trypomastigotes enter the host cells’ intact mucosal membranes (conjunctiva) or through the bite injury. Then, inside the host, the trypomastigotes infiltrate cells near the site of injury and transform to amastigotes. Subsequently, amastigotes multiply in binary fission, and they differentiate into trypomastigotes where they are released into the blood circulation (bloodstream trypomastigotes). The vectors become infected by feeding on human or animal blood that contains circulating parasites. Trypomastigotes transform into epimastigotes in the vector’s midgut where they multiply. Finally, they transform into infective metacyclic trypomastigotes and they migrate into the rectum of vector. (**c**) *Leishmania* spp. are transmitted in the saliva of the sandfly vector to humans and domesticated dogs. In the sandfly vector, the leishmanial species replicate as promastigotes and differentiate into metacyclic promastigotes. Then, the parasites are injected into the skin of the vertebrate host, and invade mononuclear cells, mainly macrophages, where the parasites differentiate to replicative amastigote forms within the parasitophorous vacuole. Upon replication, the amastigotes lyse the cells and infect other macrophages, which can be ingested by female sandflies during bloodmeal.

*Trypanosoma brucei*, *Trypanosoma cruzi*, and *Leishmania* spp. share a common, but distinct from other protozoan parasites, mitochondrial DNA, the kinetoplast, which defines their class (*kinetoplastida*) of phylogenetic order. *Kinetoplastida trypanosomatidae* protozoans have divergent evolutionary patterns from the eukaryotic mammal lineages, a fact which is reflected in their distinct biology, as shown in Figure 1. *T. brucei*, *T. cruzi*, and *Leishmania* spp. are eukaryotic parasites that exhibit a developmental stage within the gut of insect vectors and tissues of vertebrate hosts. During the vertebrate infective stages, the above protozoans alter the differential expression of virulence genes, modifying their biological and antigenic properties to subvert the host protective immune responses and establish a persistent infection. One of the characteristics of these protozoans is their evasion mechanisms from host immunity, which results in disease chronification [17].

### 2.2. Human African Trypanosomiasis

HAT is characterized as a tropical disease, since it mostly affects the population of West/Central Africa (95% of the cases) and East Africa (5% of the cases) [18]. It is also considered as one of the “Neglected Diseases”, given that the drugs that are prescribed for its treatment have not been improved since their initial circulation (1960), due to the limited market share [19].

In 1995, about 25,000 cases were detected with HAT disease, 300,000 undetected cases were estimated, and 60 million people were estimated to be at risk of HAT infection. In the ensuing years, the mortality rates for HAT have decreased substantially and fewer than 1000 cases were found in 2019 [20]. The disease has been characterized as endemic and consequently, the need for the development of a vaccine is fundamental. However, vaccination will not bring a radical solution to the HAT issue, since the parasites’ antigenic variation will possibly make the vaccine ineffective, and the disease will continue to infect thousands of people per year. In conclusion, the development of improved drugs, presenting high bioavailability and low toxicity, is crucial for the battle against HAT [14].

The main subspecies of *T. brucei* parasites using humans as a host are *Trypanosoma brucei gambiense*, *Trypanosoma brucei rhodesiense*, and *Trypanosoma brucei*. The first two are detected in West/Central Africa and East Africa, respectively, while the last is rarely spotted with the population of these regions. The parasite is transmitted to humans and domestic animals through the bite of infected tsetse flies and causes the endemic HAT disease [21].

People diagnosed with HAT suffer from fever, headaches, weakness, pain in the joints, lymphadenopathy, and stiffness. The infected population may not present these symptoms immediately, but only when the parasite has penetrated the blood–brain barrier (BBB). At this point, the parasite affects many human biological functions. The name of the disease, i.e., sleeping sickness, is attributed to its primary symptom, sleeping disorder. The central nervous system (CNS) is penetrated, causing a variety of different neuronal misfunctions, such as deep sensory disturbances, abnormal tone and mobility, ataxia, psychiatric disorders, seizures which slowly lead to coma, and finally, death. 

The duration of the disease is differentiated from *T.b. gambiense* to *T.b. rhodesiense*. The progression of the disease happens during two distinguishable stages, defined by the parasite’s penetration to the BBB. Before the parasite enters the CNS, which is considered as the first stage of the disease or the hemato-lymphatic stage, patients suffer from vague symptoms linked to a variety of diseases, such as fever, pruritus, arthralgia, enlarged lymph nodes, fatigue, and headaches, making the disease difficult to recognize. After the entrance of the parasite in CNS, there is the second or meningo-encephalitic stage, where the symptoms get more intense and specific. Finally, the presence of severe symptoms, including neuropsychiatric conditions, ultimately lead to coma and death [22].

Hence, HAT’s diagnosis and treatment should be performed during the expression of the first symptoms in patients. However, as it has been already mentioned, the first stage of HAT includes common symptoms that are also linked to many other diseases, rendering HAT’s diagnosis difficult to perform.

Consequently, HAT’s diagnosis includes the operation of laboratorial tests to people populating these regions, who present “suspicious” symptoms that could reveal the possibility of infection. The confirmation of the results derives from a three-step testing procedure. First, blood samples are taken from patients and they are screened, following the card agglutination test for trypanosomiasis’ protocol. Patients who are positive to the presence of the parasite undergo further tests to identify trypanosomes in blood and lymph node aspirate samples. The third step is identifying the stage of the disease in patients. This is the most challenging part of the procedure, since mild symptoms overlap between the two stages. Herein, samples of cerebrospinal fluid are taken from patients and cell cultures reveal the stage of their disease.

Recently, rapid diagnostic tests (RDTs) have been also developed in order to detect the presence of the same native antigen as the one used in the test process described above. Rapid tests were developed in 2016 and 2017 by two different companies and they presented high accuracy (99%), important specificity, and stability in various temperatures. Thus, RDTs are currently advantageous, offering accurate, sensitive results and they are strongly recommended for use to all primary medical facilities [23].

### 2.3. Chagas Disease

*Trypanosoma cruzi* is a hemoflagellate protozoan, transmitted mainly by blood-sucking triatomine bugs and it causes human Chagas disease (CD), also known as American trypanosomiasis [24]. It was identified in the first decade of the 20th century and it still remains a major problem for public health [25]. More specifically, Chagas disease is a neglected tropical illness from Latin America that spread through immigration and became a global issue [25]. An estimated 6 to 7 million people worldwide are infected with *T. cruzi* and 75 million people are at risk of infection [26]. CD was discovered by Brazilian researcher Dr. Carlos Chagas in 1909 [27].

Chagas disease can cause a sudden illness after 1–2 weeks (acute phase), or it may appear 10–20 years after initial infection (chronic phase). The acute phase of CD that can last a few weeks or months (4–8 weeks), is often asymptomatic. Acute phase symptoms include fever, fatigue, rash, body aches, eyelid swelling, headache, loss of appetite, nausea, diarrhea or vomiting, swollen glands, enlargement of the liver or spleen, and Romana sign (also known as chagoma: unilateral palpebral oedema when the conjunctiva is the portal of entry). Chronic phase symptoms may include irregular heartbeat, heart failure, sudden cardiac arrest, difficulty swallowing due to enlarged esophagus, stomach pain, or constipation due to enlarged colon. In both acute and chronic phases, the disease can impact multiple organ systems, leading to cardiopathy or megaviscera [25].

There are two mechanisms of Chagas infection transmission, primary (or main) and secondary [28]. Primary mechanisms include transmission by vectors (triatomines), by blood transfusion, orally (contaminated food), and across the placenta or through the birth canal [29,30]. Secondary mechanisms of transmission are: handling infected animals, organ transplantation, sexually, criminally induced infection by inoculation or orally, and laboratory accidents [31].

### 2.4. Leishmaniases

The Leishmaniases are a group of infectious diseases caused by protozoan parasites from more than twenty *Leishmania* species. The insect vector is a female phlebotomine sandfly, which transmits the parasite to mammalian hosts (humans, canines). The parasites that cause leishmaniasis are present in 98 countries around the world and more than 1 billion people live in areas endemic for leishmaniasis and is at risk of infection [29]. Leishmaniasis is widespread on all continents except Australia and Antarctica and according to WHO, 1.3 million of new cases occur annually [30]. There are three main forms of the disease: cutaneous leishmaniasis (CL), visceral leishmaniasis (VL), also known as kala-azar, and mucocuntaneous leishmaniasis (MCL). The clinical features vary depending on the parasite’s characteristics and on the genetic aspects of the host that determine the effectiveness of the immune response [32]. Dogs are considered main reservoirs of the parasite, although rodents, wild rabbits, hares, and cats can also play a relevant role as reservoir host [29].

Cutaneous leishmaniasis is the most prevalent clinical form of leishmaniasis worldwide, and 90% of all CL cases occur in only seven countries: Afghanistan, Algeria, Brazil, Iran, Peru, Saudi Arabia, and Syria. It usually produces ulcers on the exposed parts of the body, such as the face, arms and legs [33]. Psychiatric disorder is associated with patients with extensive lesions that are formed due to cutaneous leishmaniasis. These patients were found to have a low quality of life with symptoms of depression, high anxiety, and low body image satisfaction [34]. CL is the most common form, but VL is responsible for the majority of deaths from leishmaniasis. In visceral leishmaniasis (caused by *L*. *donovani* or *L. chagasi*), the parasite systematically attacks and spreads throughout the body, infecting the body’s macrophage cells, which then carry the parasite to the spleen, lymph nodes, liver, and bone marrow [35]. Of the 200,000 to 400,000 new cases of VL worldwide, more than 90% occur in six countries: India, Bangladesh, Sudan, South Sudan, Ethiopia, and Brazil. MCL is clearly distinguishable from other cutaneous leishmaniases by its chronic, latent, and metastatic behavior. In mucocutaneous leishmaniasis, the lesions can lead to the partial or total destruction of the mucous membranes of the nose, mouth, and throat cavities and surrounding tissues [33].

## 3. Current Regimen for Kinetoplastid Diseases

### 3.1. HAT Trypanocidals

Nowadays, the treatment of HAT disease includes the following five drug options: pentamidine, suramin, melarsoprol, eflornithine (administrated as monotherapies), and nifurtimox–eflornithine combination therapy (NECT) [36]. Pentamidine is considered as the first line treatment for early-stage HAT caused by *T.b. gambiense*, while *T.b. rhodiense* is treated with suramin.

Pentamidine, an aromatic diamine, was first introduced in 1940s and is considered a broad-spectrum drug, targeting parasitic worms, protozoa and fungi [36]. Apart from early-stage HAT, it has been also administrated for the treatment of leishmaniasis. Pentamidine presents relative toxicity and patients need to be monitored during therapy. This drug acts by preventing the synthesis of trypanosomal proteins, nucleic acids, phospholipids, and folate and its mechanism of action includes the inhibition of the enzymes participating in the polyamine synthesis and the RNA polymerase activity [37]. 

Suramin is one of the first successful drugs of early medicinal chemistry and it is administrated for first-stage HAT caused by *T.b. rhondesiense*. This compound, first introduced in 1917, is considered a broad-spectrum drug, presenting very promising results against other trypanosomes. Its administration for HAT disease involves five weekly intravenous injections of 20 mg/kg. Suramin’s mechanism of action towards HAT has not been established yet, even though it has been an extensively used drug for the past century. There is some evidence that suramin inhibits glycolytic enzymes of *T. brucei* that have high isoelectric points (>9), because of the presence of basic aminoacids in high ratio. The question that has been addressed but not answered yet is whether the drug penetrates the membrane and acts on the glycolytic enzymes inside the glycosomes or whether it binds firstly to the cytosolic glycolytic enzymes and then enters the glycosomes, where these enzymes reside. Apart from the inhibition of glycolytic enzymes, suramin could also prevent sleeping sickness by inhibiting glycerophosphate oxidase, serine oligopeptidase, and the RNA-editing ligase of the trypanosome’s kinetoplast, although the drug’s penetration to the mitochondrial membrane still remains unclear [38].

Eflornithine, available in the market since 2001, has replaced melarsoprol, as it is much safer to consume. Melarsoprol presented high toxicity that leads to reactive encephalopathy [39,40] followed by death. However, eflornithine requires huge doses of administration (56 infusions during 14 days in 14 liters of sterile saline) and consequently, daily visits of patients in health facilities and hospitals. This is a very difficult issue to resolve, since the accessibility to medical care facilities is considered as a privilege of big cities, in the regions that mostly suffer from HAT. Habitants of rural areas do not have easy access to hospitals; thus, the toxic but easily administrated melarsoprol is still used for HAT treatment. Moreover, apart from the difficulty in administration, eflornithine presents a high risk of resistance to patients, resulting in the possibility of untreated second-stage HAT.

The concept of combining therapies started to gain ground, as a way of optimizing drugs’ efficacy and lowering the resistance to therapy [41]. Various clinical studies have been performed using a combination of efthornithine, melarsoprol, and nifurtimox (drug administrated for the treatment of Chagas disease). All the studies including the administration of melarsoprol resulted in a high level of toxicity, while promising results were revealed for the combination of eflornithine and nifurtimox (NECT therapy) concerning the treatment of second-stage HAT caused by *Trypanosoma brucei gambiense*. In order to eliminate eflornithine’s difficulty in administration, WHO designed a medical kit, consisting of the drugs in an accessible form, and trained the health workers to implement the aforementioned drug therapy [42]. The NECT therapy does not present the same results for infection caused by *Trypanosoma brucei rhodesiense*, thus melarsoprol remains the only efficient drug for this disease.

### 3.2. Antichagastic Drugs

Treatment with antitrypanosomal drugs is indicated for acute and congenital CD, chronic disease in infants, and reactivated infections [18]. Benznidazole (BZN) and nifurtimox (NFX) have been the only two nitroheterocyclic available drugs for CD’s treatment since the 1960s [25,28,43]. These drugs are effective in the acute phase, whereas in adults with chronic infection, clinical trials have shown limitations, and both drugs cause several toxic effects leading to restrict their use [28,43]. The mechanism that results in the activation of nitroheterocyclic drugs in trypanosomes is achieved through the involvement of the nitroreductase enzyme (NTR). The resistance of BZN and NFX occurs through the reduced activity of ΝΤR [18]. Benznidazole prevents the multiplication of a parasite, covalently modifying macromolecules. Nifurtimox generates nitro radical anions and becomes reactive in the presence of oxygen [19]. Nifurtimox is no longer used as a therapeutical agent of CD in Brazil due to the fact that it causes neurological disorders or psychiatric episodes as side effects [20,21]. Many patients find out that they have contracted of Chagas disease years after the infection. Therefore, there is an urgent need to design novel, effective, and non- toxic drugs.

### 3.3. Antileishmanial Drugs

The chemotherapy currently used for the treatment of leishmaniases include a small spectrum of drugs, that are used as monotherapy or in combination. Provided that there is no vaccine available for the prevention of leishmaniases, the control of the disease depends mainly on these drugs. The therapeutic scheme depends on the type of the disease (cutaneous or visceral leishmaniasis), the parasite species, the geographic location, and probable underlying diseases of the patient. These drugs are in general repurposed from other indications and there are severe limitations concerning their use, which indicates the need of new regimen options. The significant toxicity, low efficacy, and increasing drug resistance are some of the most important problems of the current drugs. The high cost and poor availability of drugs, along with the long duration of therapy and need of hospitalization, are also major concerns, especially for countries in which leishmaniasis is endemic [13,44].

Pentavalent antimonials are the oldest category of antileishmanial drugs. The use of antimony for the treatment of leishmaniasis has been known for several centuries and the first pentavalent antimony compound was synthesized and proved effective against VL in 1925. Today, there are two pentavalent antimonials that are used as first-line antileishmanial drugs: sodium stibogluconate and meglumine antimonate. These drugs are inhibiting enzymes of the glycolytic pathway and fatty acid oxidation in *Leishmania* parasites. [44] While pentavalent antimonials are used as first-line drugs, their efficacy varies significantly depending on the country, and especially in India, where high rates of resistance are observed [45].

Amphotericin B is a polyene antibiotic which has been used as an antileishmanial drug for several decades. Amphotericin B increases the membrane permeability of *Leishmania* spp., by binding to ergosterol of the parasite membrane [43]. This drug is used as a second-line drug for VL, and even though it exhibits major side effects such as nephrotoxicity [44], it is recommended from WHO as first-line therapy in South Asia, where the cure rate of antimonial drugs is low. AmBisome^®^, a liposomal form of amphotericin B, is very effective and lacks of the side effects of the conventional amphotericin B, but its cost is unaffordable, especially for some endemic countries [46]. Paromomycin is another aminoglycoside antibiotic, which has been used as an antileishmanial agent since the 1980s and is currently administrated as a second-line drug, but it is not used as a single agent, because of the risk of developing drug resistance [44,46].

Miltefosine is an alkylphospholipid derivative, approved for the treatment of leishmaniasis in 2002. It was firstly developed as an antineoplastic agent but showed activity against *Leishmania* spp., and it is the only administrated oral treatment until now. Miltefosine modulates parasite membrane receptors and changes the structure of the cell membrane by inhibiting the metabolism of phospholipids. It also affects various intracellular pathways leading to regulated cell death [47]. It has significant toxicity and teratogenic properties and there are several restrictions on its use [44,45]. Combination therapy with at least two drugs is proposed to increase the therapeutic efficacy and avoid drug resistance. The use of a combined dosage form also aims to shorten the duration of therapy and reduce the drug doses as well as to eliminate the toxicity and increase the compliance of patients. Moreover, this therapeutical approach is cost effective for public health systems. There are several forms that are used, such as sodium stibogluconate with paromycin and amphotericin or paromycin in combination with miltefosine. There are also some repurposed drugs, e.g., pentoxyfilline, allopurinol, and various azoles that are used in combination dosage forms, along with antileishmanial drugs [44,46]. 

The current regimen against kinetoplastid diseases is depicted in Table 1.

## 4. Drug Targets and Inhibitors

### 4.1. Kinetoplastid Proteasome

Even if the identification of a common kinetoplastid drug target against *T. brucei*, *T. cruzi*, and *Leishmania* spp. looks like a unicorn of antiprotozoan chemotherapy, the kinetoplastid proteasome has recently become an attractive therapeutic target. Proteasomes are complex enzymes responsible for the turnover of short-lived, abnormal, or damaged proteins in eukaryotic cells. Kinetoplastid proteasomes resemble but distinguish those of other eukaryotes. Two separate studies have shown similar proteasome inhibitors with excellent potency and selectivity against kinetoplastid parasites, both in vitro and in vivo [48]. The Genomics Institute of the Novartis Research Foundation (GNF) has identified an azabenzoxazole compound series (GNF5343, **1** and the optimized GNF6702 derivative **2**) as pan-kinetoplastid proteasome inhibitors [49]. Moreover, GSK and Dundee Drug Discovery have also identified a similar azabenzoxazole adduct (GSK3494245/DDD01305143, **3**), which exhibits similar structural features to the GNF derivative [50]. Structural studies have revealed the binding site for both clusters of compounds in an open pocket between the proteasome subunits. The binding mode suggests a different orientation for the two-compound series, but this may need to be revised. Pyridine substitution improved the solubility of GNF6702 affording LXE408 adduct, which is now being tested in Phase I clinical trials [51]. These findings create the expectation that the kinetoplast proteasome might be the Achilles’ heel of all trypanosomids. The above chemical entities are listed in Table 2.

### 4.2. T. brucei

#### 4.2.1. RNA-Editing Ligase 1 (REL1)

Novel drug approaches aim to target the trypanosome’s survival and replication in order to prevent its spread throughout the body. A key enzyme participating in trypanosome’s survival in both insect and bloodstream form is the RNA-editing ligase 1 (REL1). Thus, REL1 is considered as a promising target for *T. brucei*’s elimination. Computational studies have been applied by Amaro et al., suggesting molecular dynamics simulations on the REL1, in order to take account of the protein’s flexibility in the drug discovery process [52]. Docking studies, following the molecular dynamic (MD) simulations, were performed to different conformations of the enzyme and revealed 30 compounds as competent inhibitors of REL1. The compounds that presented the strongest binding affinity to the protein’s active site were further evaluated through MD simulations. The final scoring pinpointed eight ligands that were examined through in vitro biological assays. In fact, two of the proposed compounds **4**, **5** indicated important selectivity and low micromolar activity against REL1 [53].

#### 4.2.2. UDP-Glucose 4′-Epimerase (GalE)

Computer-aided studies have identified Uridine Diphosphate Galactose 4′-Epimerase inhibitors as novel therapy for African sleeping sickness. TbGalE, also known as UDP-glucose 4′-epimerase, participates in galactose metabolism of the parasite *T. brucei*. Galactose is an essential component of surface glycoproteins that plays a key role in the evasion of the parasite in host cells. Thus, TbGalE could also serve as a potent target for HAT therapy. Durrant’s research group identified TbGalE inhibitors using a combination of docking and MD studies. Twenty-four clusters of the protein were identified and used as input for the docking studies. The trajectory analysis of the protein offers more chances for discovering effective ligands. The compounds that presented the strongest binding affinities in the in silico calculations of UDP-glucose were further evaluated experimentally. In vitro assays of the ligands indicated that the fused ring systems of the structures, even though they contribute to ligand binding, do not reinforce the selectivity of the compounds, thus they could possibly be replaced by an equivalent group. This study suggests taking advantage of the presence of a thiol-group in position C266 of the protein in order to ameliorate the ligands’ selectivity, because this structural characteristic (thiol-group) is absent in the corresponding protein of humans [54]. According to this investigation, compound **6** indicates the most favored inhibitory activity against TbGalE.

#### 4.2.3. Pteridine Reductase-1 (PTR1)

Another significant enzyme for trypanosome’s survival that could serve as a target for the treatment of HAT is pteridine reductase-1 (PTR1) [55]. Drugs bearing aminobenzothiazole and aminobenzimidazole scaffolds presented promising inhibitory activity against PTR1. Virtual screening of a fragment library was performed in order to discover the structural properties of potent PTR1′s inhibitors. Docking studies in PTR1′s crystal structure (PDB: 3BME) indicated the required ligands’ characteristics for the exertion of inhibitory activity. Computational studies were followed by complexes’ crystallization so as to discover the most accurate binding mode of the ligands inside PTR1′s cavity. Furthermore, among all the compounds that were tested, compound **7** was evaluated as a selective inhibitor with low micromolar potency [56].

#### 4.2.4. Histone Deacetylase Enzymes (HDAC)

Several anticancer therapies have been investigated for their use against parasitic diseases. According to Murphy et al., CTLA-4 blockers enhance host resistance against Leishmania donovani [57], while the same effects have been observed for PDL-1 blockers by Habib et al. [58]. Histone deacetylase enzymes (HDAC) play a significant role in the modulation of parasites’ gene expression, and hence they could serve as potent targets for HAT therapy [59]. Many inhibitors of these enzymes (class I/II HDAC inhibitors and sirtuin inhibitors) have been discovered and approved for anticancer therapy. Engel et al. identified four HDAC inhibitors that presented important inhibitory activity towards trypanosomal parasites. Even if these compounds could effectively inhibit HDAC enzymes (cmpd **8**), they exerted significant cytotoxicity on the cell lines. The most potent compounds for HDAC inhibition proved to be sulphonylpiperazines and in particular compound **8** (IC_50_ = 34 nM), which bears an heteroaryl ring attached to the piperazine scaffold. This adduct not only caused parasitic death within 4 h after treatment, but also its dosage for administration was quite low (2 μg/mL). Furthermore, hydroxamate-based HDAC inhibitors were tested for their potency to inhibit the proliferation of *T.b. brucei* in the bloodstream. These are clinically approved anticancer agents that indicated moderate potency against the parasites. Unfortunately, these derivatives were further tested as parental compounds for co-administration with standard treatment (pentamidine, suramin, melarsoprol, nifurtimox), with no promising results for antiparasitic activity [60]. 

#### 4.2.5. *N*-Myristoyltransferase (NMT)

A popular enzyme that can be targeted for the treatment of HAT is *N*-myristoyltransferase (NMT) [61,62,63]. High-throughput screening was performed against NMT and generated a series of compounds that are active in a mouse model of the first stage HAT. NMT inhibitors cause the death of trypanosomes both in mice and cell lines, by binding strongly to NMT’s cavity and inhibiting the enzyme’s function. The only problem is that NMT also exists in humans, although this problem could easily be resolved since *T. brucei* indicated more sensitivity towards the protein’s inhibition. NMT also served as a target for the treatment of second stage HAT. The treatment of the disease in the second stage is quite challenging, since the drug needs to penetrate the BBB without causing any toxicity to the cells [64].

#### 4.2.6. Trypanothione System—Trypanothione Reductase (TR)

**Figure 2:**
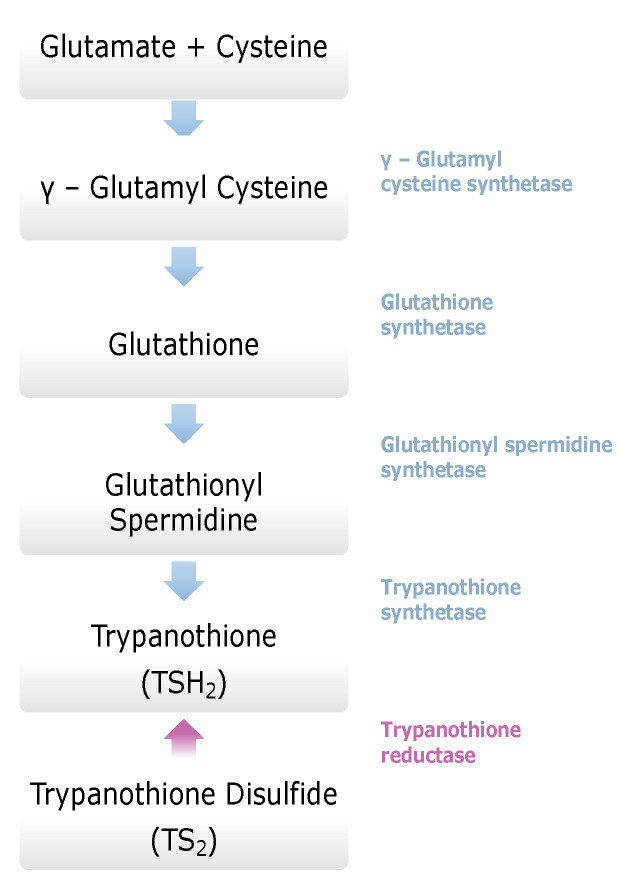
Trypanothione biosynthesis pathway.

The trypanothione system is central for any thiol regeneration in trypanosomatids. The absence of trypanothione metabolism from the mammalian cells makes TR a promising target for the development of novel, potent antitrypanosomal drugs. Several existing drugs interact with the trypanothione system (Figure 2). Specifically, three enzymes, glutathionyl spermidine synthetase, trypanothione synthetase, and trypanothione reductase, emerge as main targets of rational drug design [65]. Trypanothione reductase (TR) is a flavoenzyme which is of interest for its essential role in controlling the redox homeostasis of *T. cruzi*. Its crystal structure was solved in complex with NADPH or inhibitors or alone [66,67,68]. TR is a homodimer that includes an FAD-binding domain, an NADPH-binding domain, and an interface domain. The enzyme catalyzes the NADPH-dependent reduction of dithiol trypanothione, representing high selectivity [69].

The binding mode of the inhibitors under study varies between *T. cruzi* and *T. brucei*. Docking studies revealed that the inhibitors bind to the hydrophobic “wall” of TR’s active site. The crystallization of *T.b.* protein with BTCP (1-(1-(benzo[b]thiophen-2-yl)cyclohexyl)piperidine) inhibitor in the active site led the drug design investigation, conducted by Persch et al. BTCP analogues were designed in silico and further assessed by docking studies. The binding pocket “Z-site” was described by the sidechains of Phe396′, Pro 398′, and Leu399 and the PDB code: 1BZL was used for these calculations. The analogues that presented strong binding to the enzyme’s cavity were synthesized and enzymatically evaluated. Among all the structures that were generated, the most favorable uncompetitive inhibition constant (Kui) was calculated for derivative **7** against *T. brucei*. According to this study, the disubstituted methyl thiazole structural part is crucial for exerting antitrypanosomal activity [70]. 

Additional in silico studies were performed for more BTCP analogues to TR enzyme (PDB:1BZL) by Raoul De Gasparo et al. This study revealed new structural features of TR inhibitors. The addition of one indole group at the structural skeleton indicated improved activity against the TR enzyme of *T. cruzi*, while the addition of both benzyl and indole groups presented the strong inhibition of TR in *T. brucei* [67].

**Figure 3:**
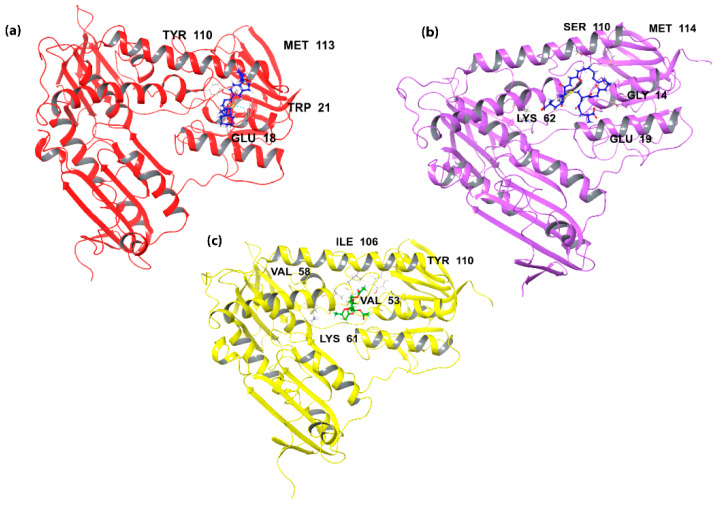
Trypanothione crystal structures with co-crystallized ligands. The residues of all the active sites are indicated in each figure. (**a**) *T. brucei* trypanothione (**b**) *T. cruzi* trypanothione (**c**) *L. infantum* trypanothione.

Finally, another study oriented towards the drug discovery of TR inhibitors was conducted by Patterson et al. in 2009. According to this investigation, cell-based enzymatic assays were performed for a variety of BTCP analogues and the compounds that presented low micromolar activity against TR enzyme were revealed [63]. The crystal structure of trypanothione with co-crystallazed ligands is illustrated in Figure 3.

The abobe novel chemical agents against *T. brucei* are presented in Table 3.

### 4.3. T. cruzi

#### 4.3.1. Ergosterol Biosynthesis Pathway

**Figure 4:**
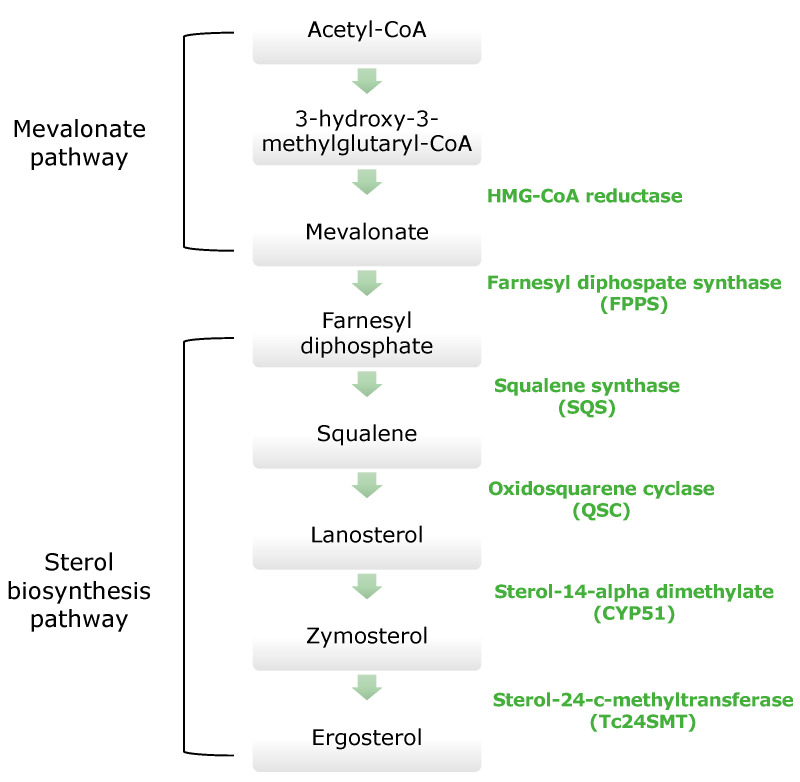
Ergosterol biosynthesis pathway.

Sterols are constituents of the cellular membranes that are vital for their structure and function. In mammalian cells, the main sterol is cholesterol, whereas in members of the *Trypanosomatidae* family, the main sterol is ergosterol [71]. Cholesterol and ergosterol have minor structural differences. An important metabolic pathway in *T. cruzi* is the sterol biosynthesis (SB) pathway, shown in Figure 4. The products of this pathway are ergosterol and other 24-sterols, which are essential for parasite multiplication. Therefore, the ergosterol biosynthesis pathway is a promising target for drug development as the survival of *T. cruzi* is dependent on sterols production [72,73]. 

Furthermore, the inhibition of enzymes of the ergosterol biosynthesis pathway plays a crucial role in antitrypanosomal drugs development. More specifically, Squalene synthase (SQS), Oxidosqualene cyclase (OSC), Sterol 14-alpha demethylase (CYP51) and Sterol 24-c-methyltransferase (Tc24SMT) are important targets for novel drug development [71,74]. 

##### Sterol 14-α-Demethylase (CYP51)

The *T. cruzi* sterol 14-alpha demethylase (CYP51) is a crucial enzyme for parasite survival. The enzyme is responsible for catalyzing the conversion of lanosterol to zymosterol. CYP51 has been found as a significant target for the development of antitrypanosomal, antileishmanial, and antifungal drugs. Azoles were identified as a new potent class of inhibitors. Specifically, bifonazole (**12**), clotrimazole (**13**), econazole nitrate (**14**), miconazole (**15**), and tioconazole (**16**) as imidazoles as well as itraconazole (**17**) and ketoconazole (**18)** as triazoles demonstrated high IC_50_ values (IC_50_ = 0.003–0.3 μM) as CYP51 inhibitors [75]. Molecular docking and in vitro studies showed that arylsubstituted derivatives could act as antiprotozoal agents in the active site of CYP51. The ligand **19** was found to exhibit no toxicity according to the study conducted with the MRC-5 cell line and the best selectivity index against *T. cruzi*, *T.b. brucei*, and *T.b. rhodesiense* [76].

#### 4.3.2. Trypanothione Reductase (TR)

As it has already been described (Section 4.2.6), the TR enzyme is an attractive target for antitrypanosomal drugs. Clomipramine (**20**), a tricyclic antidepressant, has been shown to be active against infected mice in the chronic cardiac phase of CD, as it lessened cardiac damage [72]. Quinoxaline derivatives showed antitrypanosomal activity by inhibiting TR [8]. In silico studies showed that quinoxaline-7-carboxylate 1,4-di-*N*-oxide derivatives **21**, **22**, and **23** exhibit high anti-*T. cruzi* activity. Derivative **21** and **22** presented higher values of lysis on both strains of *T. cruzi* than Nfx and Bzn [73]. In silico studies of Arguelles et al. proposed quebrachamine (**24**), cephalotaxine (**25**), cryptolepine (**26**), (22S,25S)-tomatidine, (22R,25S)-solanidine, and (22R,25R)-solasodine as novel alkaloid scaffolds for the development of novel and potent TR inhibitors [77].

#### 4.3.3. Enolase

Enolase (2-phospho-D-glycerate hydrolase) has an essential role in glycolysis and glycogenesis. It is a metalloenzyme that catalyzes the reversible dehydration of D-2-phosphoglycerate (PGA) to phosphoenolpyruvate (PEP). It is found in a variety of organisms, from bacteria to mammals, including trypanosomatids [78]. Enolase requires magnesium for both catalysis and dimer stabilization. Additionally, enolase acts on the cell surface of pathogens as a plasminogen receptor [71]. Consequently, enolase represents an attractive target for the development of drugs against *T. cruzi*. A combination of computational studies concluded that etidronate could be a promising drug of Chagas disease as TcENO inhibitor, since it presented high binding energy and low RMSD values [79]. Etidronate (**27**) is a drug currently used in the treatment of Paget’s disease, osteoporosis, and heterotropic ossification with rare side effects [80].

In silico and in vitro studies demonstrated that butenolides could present interesting scaffolds for the synthesis of derivatives with potent activity against *T. cruzi.* Biological studies have showed that isolated compounds from *Nectandra* established antibacterial activity and parasitic infection [81]. Particularly, isolinderanolide D (**28**) and isolinderanolide E (**29**) were isolated from *Nectandra oppositifolia*. These two compounds were effective against the trypomastigote and amastigotes forms of *T. cruzi*, without any mammalian cytotoxicity [82].

#### 4.3.4. Cruzain (Cz)

Cruzain or Cruzipain (Cz) is the major papain-like cysteine protease of *Trypanosoma cruzi*. Cz is vital for the survival and the multiplication of this parasite [83]. The enzyme contains a catalytic domain and a C-terminal extension [84]. Vinyl sulfone (K777) **30** was the representative candidate cruzain inhibitor [85]. K777 is a peptide derivative which binds irreversibly to cruzain while its oral bioavailability was low, and its half-life was short. Additionally, pre-clinical studies using K777 identified that this drug candidate was highly hepatotoxic [86]. Yepes et al. suggested chalcones–quinoline conjugates as potential inhibitors of *T. cruzi* Cz. The core structure of chalcones has been used as an effective scaffold for the development of antichagastic drugs [87]. The quinoline moiety also presents biological properties and is active against *T. cruzi* by inhibiting cruzain. The results of this study demonstrated that the most active chalcones–quinoline conjugates displayed promising in vitro inhibition (EC_50_ values range between 7.0–36 μM) in comparison with Bnz (EC_50_: 40.3 ± 6.92 μM). The combination of computational approaches showed that compounds **31** and **32** had the optimal drug-like properties [88]. Computational studies also demonstrated that thiophen-2-iminothiazolidine derivatives could be used as cruzain inhibitors. Many thiophene compounds have been reported for their activity against *T. cruzi*. Thiazolidine compounds have recently become a medicinal target. The most active derivative against cruzain was thiophene–thiazolidine hybrids **33** with IC_50_ values of 2.4 μM [89].

#### 4.3.5. Ribose 5-Phosphate Isomerase Enzyme (Rpi)

The ribose 5-phosphate isomerase (Rpi) enzyme plays a key role in the pentose phosphate pathway (PPP). It is responsible for the production of nucleotide precursors and NADPH. Rpi catalyzes the reversible isomerization reaction between *D*-ribose-5-phosphate (R5P) and *D*-ribulose-5-phospate (Ru5P) [90]. The inhibition of Rpi enzyme is an attractive target for drug development against *T. cruzi*. Molecular docking and molecular dynamic simulations revealed that two compounds, ZINC36975961 and ZINC43763931, had good performance and made interactions with key residues of the active site for the total simulation time. The compounds ZINC36975961 and ZINC43763931 are potential inhibitors of *T. cruzi* Rpi [80].

#### 4.3.6. Isocitrate Dehydrogenase 2 (IDH_2_)

Two isocitrate dehydrogenases (IDH_1_ and IDH_2_) are displayed in *T. cruzi.* IDHs enzymes catalyze the oxidative decarboxylation of isocitrate, producing 2-oxoglutarate, CO_2_ and NADH or NADPH [91]. Sertraline (SERT) (**34**), a serotonin reuptake inhibitor, revealed antitrypanosomal activity against *T. cruzi*. Homology modeling and molecular docking studies showed that the presence of a hydrophobic pocket could provide hints for the structural optimization of sertraline in order to design novel TcIDH_2_ inhibitors [92]. 

#### 4.3.7. Dihydrofolate Reductase–Thymidylate Synthase (DHFR-TS)

Dihydrofolate reductase–thymidylate synthase (DHFR-TS) is a bifunctional enzyme that catalyzes the reduction of folate and the subsequent synthesis of thymidylate in DNA synthesis in *T. cruzi* [93]. According to Juárez-Saldivar et al., DHFR-TS could serve as a useful target to apply drug discovery against Chagas disease. In this study, Juárez-Saldivar’s research group has developed a strategy which combines docking studies and the statistical analysis of protein–ligand interactions so as to design compounds with strong inhibitory activity. FDA-approved drugs and food compounds were evaluated as potent DHFR inhibitors and the most prominent were evaluated in vitro. Biological assays pinpointed compound **35** as a novel therapeutic agent against Changas disease [94].

Another computational study, conducted by Schormann et al., identified the pharmacophore structure that derived from 3D QSAR studies of a ligand library to the DHFR-TS enzyme. All ligands in the library contained the 2,4-diamino-pyrimidine substructure which is characteristic for DHFR inhibitors, and they were crystallized with DHFR. This study revealed that the binding pocket of the enzyme is almost exclusively hydrophobic and the binding affinity of the ligands depends on the structure of the ring fused to the 2,4-diamino-pyrimidine substructure **36** [95]. 

A new protocol called mt-QSAR-MLP uses QSAR for the application of multi-target drug discovery against parasitic diseases and aims to predict inhibitors that target proteins of various parasites, simultaneously. This protocol afforded derivatives, e.g., compound **37** that successfully inhibit DHFR-TS, as well as enzymes of other parasites [96].

#### 4.3.8. Pteridine Reductase

Pteridine reductase, an essential enzyme for the survival of *T*. *cruzi*, could also serve as a potent target for drug design against Chagas disease. Nine quinazoline derivatives were synthesized by Mendoza-Martínez’s research group and evaluated as potent anti-trypanosomatid agents. Docking studies have been performed to pteridine reductase, followed by in vitro biological evaluation of the compounds. Quinazoline derivatives **38** presented low micromolar activity against pteridine reductase of *T. cruzi,* while three of the compounds under study exhibited better results than Nfx and Bzn, which were used as reference drugs [97].

#### 4.3.9. Farnesyl Diphosphate Synthase (FPPS)

Farnesyl diphosphate synthase (FPPS) is an enzyme that catalyzes the condensation of isopentenyl diphosphate with dimethylallyl diphosphate to farnesyl diphosphate (FPP) in *T. cruzi.* Eight metal complexes were evaluated by Demoro et al. in order to evaluate their potency to serve as FPPS inhibitors. Biophosphonates, alendronate and pamidronate (pam), which complexed with the following metals: Cu, Co, Mn, and Ni, were crystallized and evaluated for their activity against FPPS of *T. cruzi*. These metal complexes presented strong inhibition against FPPS of *T. cruzi* in contrast to FPPS in mammalian cells. A representative complex **39** is illustrated in Table 4 [98].

#### 4.3.10. Sirtuins

Silent information regulator 2 (Sir2) or sirtuin inhibition could be an attractive target for drug development against CD. There are two sirtuins in *T. cruzi*: TcSir2rp1 and TcSir2rp3. TcSir2rp1 is localized in the cytosol and TcSir2rp3 in the mitochondrion. Sirtuins are NAD^+^-dependent enzymes, which are responsible for many cells functions such as gene silencing, DNA damage repair, and several metabolic processes. Sirtuins catalyze the deacetylation of lysine in the presence of NAD^+^-producing nicotinamide and *O*-acetyl-ADP-ribose [99]. The studies of Bastos et al. focused on derivatives of cardol, cardanol, and anacardic acid. Their results demonstrated that natural compounds isolated from cashew nut (Anacardium occidentale, *L. Anacardiaceae*) presented anti-*T. cruzi* activity by inhibiting sirtuin [100].

The new antichagastic agents are listed in Table 4.

### 4.4. Leishmania spp

#### 4.4.1. Purine Salvage Pathway

*Leishmania* parasites do not present the essential enzymes for the de novo biosynthesis of purines and they exhibit retrieving mechanisms to obtain the purines from the mammalian host cells [34]. The purine salvage systems of the parasites consist of unique transporters that might be drug targets, even if it is not easy to be selectively inhibited. Phosphoribosyltransferases (PRTs) play a crucial role in the purine salvage pathway [44] and more than one PRT must be targeted to successfully inhibit the biosynthesis of purines, because of the existence of alternative pathways for the retrieval of purines. LdNT1/2 (*Leishmania dovani* Nucleotide Tranpsporters 1/2) are also druggable targets, since they transfer nucleoside analogs that inhibit cell growth. Immucillins **40**, **41** are nucleoside analogs that inhibit the nucleoside hydrolase (NH36), which belong to purine salvage enzymes [101].

Nucleoside diphosphate kinases (NDKs) are significant in the purine salvage pathway. A pyrrole–indolinone adduct SU11652 **42** binds to NDKs of *Leishmania* spp. and generates a new series of NDK inhibitors with antileishmanial activity [102]. In contrast to purines, pyrimidines are synthesized de novo or through the pyrimidine salvage pathway. Consequently, 5-fluorouracil (**43**) and cytarabine (**44**) pyrimidine analogs inhibit the corresponding enzymes of the folate pathway and exhibit antiproliferative activity against promastigotes and intracellular amastigotes of *Leishmania* parasites [34,103].

#### 4.4.2. Mitochondrial Electron Chain and Cytochromes

The electron transport chain is crucial for the growth of the *Leishmania* parasites into the host cells. Blocking the electron transport chain reduces pathogen survival because of the ROS that produce toxic byproducts into eucaryote cells [104]. The enzyme that catalyzes the electron transfer from NADH to ubiquinone is known as type 2 NADH dehydrogenase and is a promising molecular target. A 6-methoxyquinalidine derivative **45** was proven as a potent inhibitor for *L. infantum* NADH dehydrogenase (LiNDH2), exhibiting antileishmanial activity in a nanomolar range [105]. Oleanolic acid (**46**) also acts on the electron chain as it interacts with CYP51 and inhibits promastigote and amastigote forms of *L. infantum.* Consequently, triterpenoids, such as oleanolic acid, are plausible antileishmanial agents [106].

#### 4.4.3. Polyamine Biosynthesis Pathway

Polyamines such as putrescine, spermidine and spermine are vital for the differentiation of *Leishmania* parasites and cell survival. There are several enzymes that are involved in the biosynthesis of polyamines and might be considered as molecular targets [44]. Arginase converts *L*-arginine to *L*-ornithine and urea and is one of the most important enzymes of the polyamine biosynthesis pathway. A shortage of arginase in the extracellular environment of *Leishmania* spp. provokes apoptosis. The benzimidazole analog **47** binds into the catalytic pocket of arginase exhibiting inhibitory activity and becomes a promising lead compound in the antileishmanial drug design [104].

The polyamine spermidine, along with two glutathione molecules, composes trypanothione, which is the only compound of trypanosomes that does not exist in mammalian cells. Therefore, enzymes that are involved in the synthesis and metabolism of trypanothione should be targeted by selective antileishmanial drugs. Trypanothione reductase (TR) is considered as one of the most important targets of the trypanothione pathway, and many studies reveal several compounds **48**, **49**, **50** that act as inhibitors [34,44,104]. The crystallographic structure of trypanothione reductase led to the identification of several classes of inhibitors that bind on specific sites of the enzyme [107]. Acridine derivative **51** binds to the active cavity (mepacrine binding site/MBS) of TR and acts as inhibitor. 3,4-Dihydroqyinazoline analogs of **52** also inhibit TR, binding to MBS. Derivatives of 1-(1-(benzo[b]thiophen-2-yl)cyclohexyl)piperidine (BTCP) are also promising competitive inhibitors of TbTR, due to their drug-like properties, such as low molecular weight and blood–brain barrier crossing. Several structural modifications of the BTCP scaffold led to indole–thiazole derivative **53**, which targets a hydrophobic sub-pocket near the catalytic cysteines in the TR active site [107]. 

Metalloid-based drugs, such as pentavalent antimonials, are currently used to treat leishmaniasis, despite having severe side effects and resistance phenomena. It is known that these drugs, at least in part, act on TR by binding catalytic cysteines. Antimonial derivatives of **54** exhibited antileishmanial activity against amastigotes, effecting the binding pocket of trypanothione reductase. Besides antimony, silver and gold were proven to bind TR in a similar way but even more efficiently. Auranofin (**55**), a gold-containing antirheumatic tested on leishmanial TR and parasites, was found to be 10-fold more potent than Sb on TR. Apart from the Au complex, the thiosugar moiety of auranofin binds the inner part of the TR catalytic site, resulting to a double inhibition mechanism [107,108]. 

#### 4.4.4. Folate Metabolism

Trypanosome parasites are folate and pterin auxotrophs, as they rely completely on pteridine salvage from their hosts. The key enzyme of folate metabolism is dihydrofolate reductase (DHFR), a target for the antifolates. Antifolates are exploited to treat several diseases such as malaria, bacterial infections, and various cancer types. An upcoming problem for the use of antifolates as trypanocides is parasite resistance to these agents. Pteridine reductase 1 (PTR1) is unique in these parasites and provides a bypass for DHFR inhibition. Since trypanosomatids are able to overcome DHFR inhibition by overexpressing the pteridine reductase 1, the inhibition of both PR1 and DHFR might provide a new therapeutical target [104,109]. Quinolone derivatives of **56** bind on the active site of PTR1, acting as inhibitors. Chromenone derivatives of **57** form a new class of PTR1 inhibitors, forming various interactions with amino acid residues of the active site. Phenyl-linked oxadiazole–phenylhydrazone hybrids of **8** are also potent PTR1 inhibitors, acting against the promastigote form of *Leishmania* spp. A series of analogs of compounds **59,** bearing a triazole ring fused with pyrazine moiety, showed significant antileishmanial activity by inhibiting PTR1 [110,111,112,113].

#### 4.4.5. Protein Kinases

Protein kinases are a promising target for antileishmanial drugs as they play an important role in the cell cycle of *Leishmania* spp. Cyclic-dependent kinases (CDKs) are an evolving drug target and CRK3 is the functional homologue of CDK1 in *Leishmania*. CRK3 is active in both amastigote and promastigote stages of *Leishmania* parasites and participates in all cell division processes. Two chemical classes, 2,6,9-trisubstituted purines of **60** and indirubines of **61**, have been identified as efficient inhibitors of CRK3 [44,114]. Mitogen-activated kinases (MAPKs) have been characterized and studied widely in *L. Mexicana*. They have an important role in the intracellular survival and infectivity of the parasite. The *L. mexicana* MAPK3 (*Lmx*MPK3) was found to be involved in the regulation of flagellar length in promastigotes and can be a possible drug target. Flavonoids, such as genistein (**62**) and chrysin (**63**), proved to be MPK3 inhibitors and represent a new series of phytochemicals against leishmaniasis. Genistein also has antioxidant properties and eliminates DNA damage caused by classic antileishmanials, such as meglumine antimonate [115,116].

The novel antileishmanial agents are summarized in Table 5.

### 4.5. Targeting Death Mechanisms of Kinetoplastid Protozoans

Regulated cell death (RCD) in kinenoplastids is a precise biochemical and molecular processes involved with regulatory steps which are still unknown. This limitation points out the controversy of using the term RCD and therefore makes the term “apoptosis-like” more appropriate. The “autophagic cell death” is also employed when the degradation of damaged structures, macromolecules or organelles is carried out through autophagy [120]. The molecular machinery of RCD comprises various subroutine forms (apoptosis, autophagy, necroptosis, etc.), which might uncover new therapeutic targets [121]. Various RCD types are impaired in the parasite metabolic pathways during differentiation processes of their life cycle. For instance, autophagy has been reported during L. Mexicana differentiation of metacyclic promastigotes into amastigotes [122]. The identification of parasite-specific regulators of RCD represent an attractive target for rational drug design for these protozoan infections [123].

### 4.6. Future Perspective of Drug Discovery

Over the last decade, there are many examples of applications of machine learning (ML) and artificial intelligence (AI) regarding NTDs [124]. As the estimated cost of developing new drugs increases and problems such as significant toxicity, low efficacy, and emerging resistance of the current regimen against NTDs remain unresolved, ML and AI are emerging as a new approach to overcome these challenges [125]. 

ML and AI have improved significantly in recent years due to rapid growth in computer-processing power, the development of advanced algorithms and the tendency to be implemented in R&D. ML is the study of computer algorithms which is automatically improved through the experience and use of scientific data, and AI is the ability of a digital computer to perform tasks with intellectual processes that characterize humans such as logic, learning, meaning discovery, and experience acquisition [126]. Chemical and biological information has become more and more available in public databases, offering new strategies in biomolecule structure prediction, mechanism of action elucidation, inverse drug design interpretation, organ or issue targeting of compounds, derivative properties, and multiobjective optimization. ML algorithms and AI can be used in a variety of applications of drug research and development and improve the current process, which is described by the failure of 9 out of 10 candidate therapies between Phase I clinical trials and regulatory approval [127].

There are plenty examples of AI/ML applications against kinetoplastid protozoan infections. ML chemoinformatics utilize high-throughput screens of the Broad Institute against *T. cruzi* and highlight active in vivo molecules [125]. Public databases such as TriTrypDB, TDR Targets, the Crystal Structures Database, and the *T. cruzi* RNA Sequence Database (in progress) provide a wealth of information that can be analyzed and validated by AI/ML methods. These approaches screen millions of compounds to predict those that bind and potentially inhibit protein function of *T. cruzi* and further present proper PK/PD properties to enter clinical trials. DND*i* and other PDPs are funding these activities to procced on the scale required for drug discovery. DND*i* is currently collaborating with Atomwise Inc., to advance drug discovery applying AI [128].

The new approaches overcome toxicity and resistance, the major drawback of the current regimen against the NTDs, focusing on specific proteins of the parasites. For instance, Jamal et al. analyzed a dataset of 292,470 available compounds of PubChem as pyruvate kinase inhibitors, using four classifier algorithms: Naïve Bayes (NB), Random Forest (RF), J48 and Sequential Minimization Optimization (SMO). The correlation of the aforementioned method scores with the docking method results predicted more possible active derivatives from a very large dataset and accelerated the discovery of new chemical entities against NTDs [129]. ML techniques have been used in combating drug resistance which has emerged in NTD pathologies. It is known that protein transporters (P-gp, ABC transporter, etc.) play a critical role in the decreased uptake or increased efflux of the drug from the parasites and generate related drug resistance [130]. Computer approaches identified parasite-specific motifs of the responsible protein transporters in *Leishmania major* and facilitated the allosteric modulation of miltefosine transporter and subsequently reduced the parasite resistance [131]. 

The progress of data science, ML and AI, and their applications in biosciences is a near future perspective. ML and AI are still in ongoing progress and their limitations concerning the data validation might influence the quality of their models to generate novel chemical space and effective drug design. This danger of irrelevant endpoints and targets will be optimized in due course by the increasing use and the development of successful achievements. Challenges and limitations should be wisely carried out and draw a path for improvement in the field of drug design and chemotherapy of NTDs, without biased and distorted results.

## 5. Conclusions

WHO has prioritized the intervention against the NTDs as a cornerstone of the vision of universal health coverage. While the course of the current COVID-19 pandemic remains uncertain, many programs and activities against the NTDs have been disrupted or delayed. This hindrance will impact on the gradual rise of these diseases towards the pre-intervention level. Remedial action should prompt an opportunity of innovation as a catch-up policy [132]. The application of scientific innovation offers new approaches against NTDs. The drug design and development against kinetoplastid protozoan infections should be accelerated over the next decade. The unmet need for new effective and safe drugs should lead the change for the treatment for trypanosomid diseases. The lack of well-validated trypanosomatid molecular targets is a drawback that should be overcome. The challenge that remains for drug discovery is the development of completely new classes of therapeutic agents with reduced host toxicity and improved administration regimens. The countdown to 2030 has begun, according to the General Director of WHO.

## Figures and Tables

**Table 1:** Current drugs for kinetoplastid diseases.

Drug	Structure	Comments [14,45]	Efficacy [45]
Chagas disease			
Benznidazole	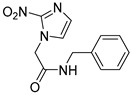	p.o. Tolerability, toxicity, incompliance.	60–85% in acute disease. Ineffective in the chronic stage.
Nifurtimox	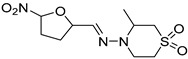	p.o. Tolerability, toxicity, incompliance.	Effective against acute disease.
Human African Trypanosomiasis (HAT)			
Suramin	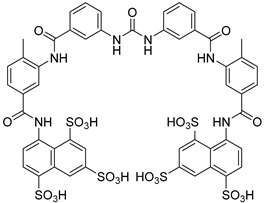	i.v. Toxicity.	Effective against hemolymphatic stage of Rhodesian trypanosomiasis.
Pentamidine	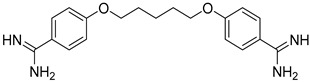	i.m. Toxicity.	Effective against hemolymphatic stage of Gambian trypanosomiasis only.
Melasoprol	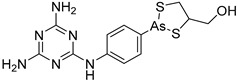	i.v. Suitable for second-stage disease. High toxicity. High levels of treatment failure in some regions.	Effective against meningoencephalic stage of both forms of the disease. It is used only for Rhodesian trypanosomiasis.
Eflornithine	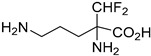	i.v. Suitable for second-stage disease. High cost. Not efficacious against *T.b. rhodesiense*.	Most effective against meningoencephalic stage of Gambian trypanosomiasis. Effective in monotherapy or in combination with nifurtimox.
NECT	Eflornithine-Nifurtimox combination	Suitable for second-stage disease. Reduced cost and duration compared to monotherapy.	
Leishmaniases			
Sodium stibogluconate	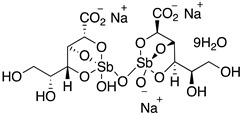	i.v. or i.m. First-line treatment. High resistance in some regions of India. Toxicity.	35–95% depending on country, resistance in India.
Meglumine antimonate	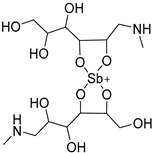	i.v. or i.m. First-line treatment. High resistance in some regions of India. Toxicity.	35–95% depending on country.
Amphotericin B	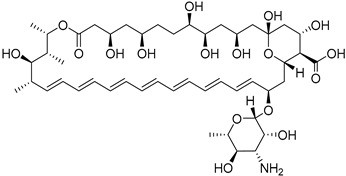	i.v. Very effective in regions with resistance to pentavalent antimonials. High toxicity.	~100% for VL in India.
AmBisome^®^	liposomial formula of amphotericin B	i.v. Well tolerated. High cost.	~95% in India and Asia, variable in Africa.
Paromycin	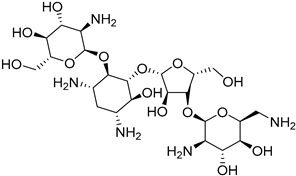	i.m. Ototoxicity.	93–95% in India, 65–85% in Africa.
Miltefosine	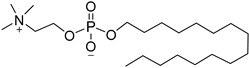	p.o. Very high efficacy for VL. Teratogenic. Increasing treatment failures.	94–97% for VL in India, not efficient as single dose in Asia/Africa, not registered in many endemic countries.

p.o.: per os, i.v.:intravenous infusion, i.m.: intramascular injection.

**Table 2:** Kinetoplastid proteasome inhibitors.

Cmpd	Structure	Name	Ref.
**1**	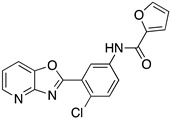	GNF5343	[49]
**2**	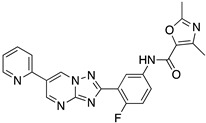	GNF6702	[49]
**3**	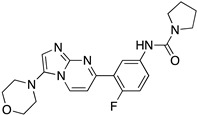	GSK3494245 DDD01305143	[50]

**Table 3:** Targets and related agents against HAΤ.

Cmpd	Structure	Target	Ref.
**4**	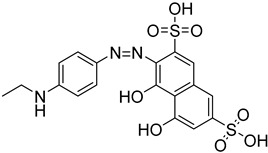	REL1	[53]
**5**	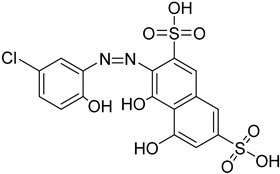	REL1	[53]
**6**	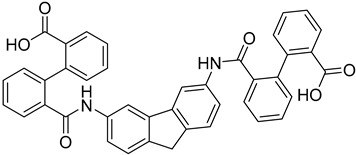	TbGalE	[54]
**7**	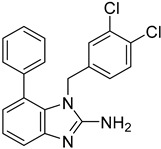	PTR1	[56]
**8**	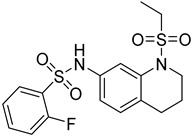	HDAC	[60]
**9**	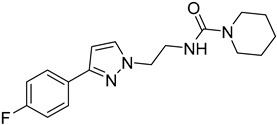	NMT	[64]
**10**	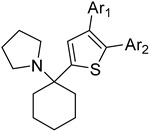	TR	[70]
**11**	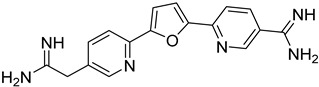		[70]

**Table 4:** Targets and related agents against CD.

Cmpd	Structure	Target	Ref.
**12**	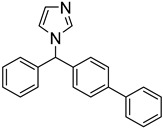	Sterol-14-a-dimethylase (CYP51)	[75]
**13**	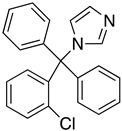	Sterol-14-a-dimethylase (CYP51)	[75]
**14**	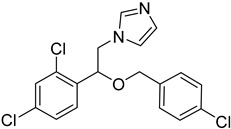	Sterol-14-a-dimethylase (CYP51)	[75]
**15**	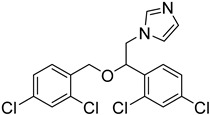	Sterol-14-a-dimethylase (CYP51)	[75]
**16**	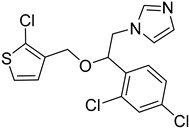	Sterol-14-a-dimethylase (CYP51)	[75]
**17**	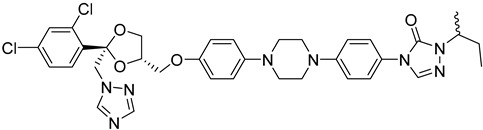	Sterol-14-a-dimethylase (CYP51)	[75]
**18**	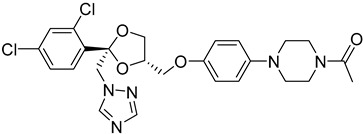	Sterol-14-a-dimethylase (CYP51)	[75]
**19**	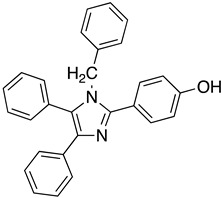	Sterol-14-a-dimethylase (CYP51)	[76]
**20**	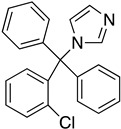	Trypanothione Enzyme (TR)	[72]
**21**	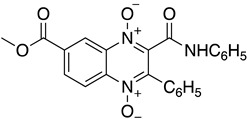	Trypanothione Enzyme (TR)	[73]
**22**	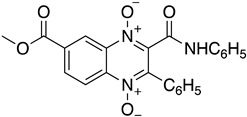	Trypanothione Enzyme (TR)	[73]
**23**	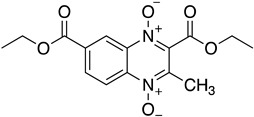	Trypanothione Enzyme (TR)	[73]
**24**	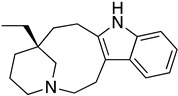	Trypanothione Enzyme (TR)	[77]
**25**	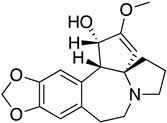	Trypanothione Enzyme (TR)	[77]
**26**	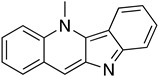	Trypanothione Enzyme (TR)	[77]
**27**	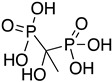	Enolase	[80]
**28**	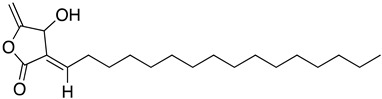	Enolase	[82]
**29**	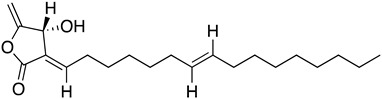	Enolase	[82]
**30**	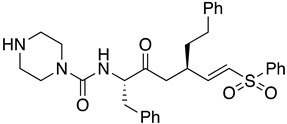	Cruzain (Cz)	[86]
**31**	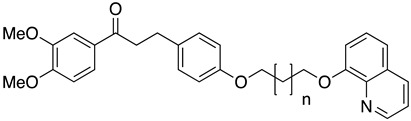	Cruzain (Cz)	[88]
**32**	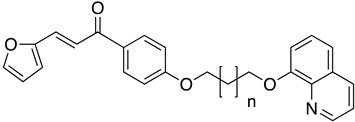	Cruzain (Cz)	[88]
**33**	** 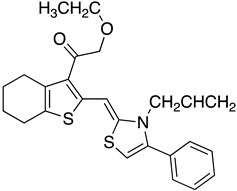 **	Cruzain (Cz)	[89]
**34**	** 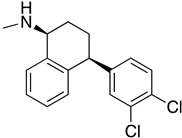 **	Isocitrate dehydrogenase 2 (TClDH2)	[92]
**35**	** 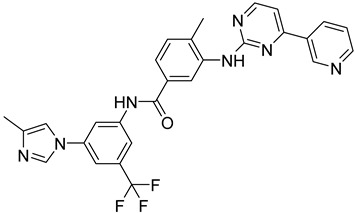 **	Dihydrofolate Reductase-thymidylate synthase (DHFR-TS)	[92]
**36**	** 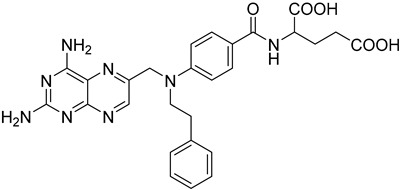 **	Dihydrofolate Reductase-thymidylate synthase (DHFR-TS)	[95]
**37**	** 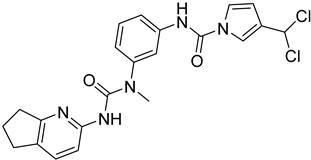 **	Dihydrofolate Reductase-thymidylate synthase (DHFR-TS)	[96]
**38**	** 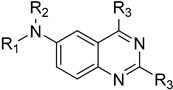 **	Pteridine reductase	[97]
**39**	[Co(Pam)_2_(H_2_O)_2_]	Farnesyl diphosphate synthase	[98]

**Table 5:** Targets and related agents against *Leishmaniases*.

Cmpd	Structure	Target	Ref.
**40**	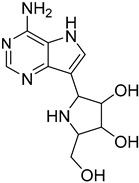	Nucleoside hydrolase NH36	[101]
**41**	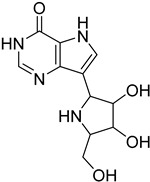	Nucleoside hydrolase NH36	[101]
**42**	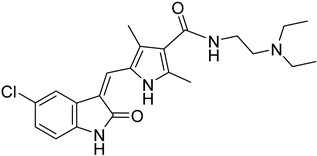	Nucleoside diphosphate kinase NDK	[102]
**43**	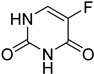	Nucleoside transporter 1 NT1	[34]
**44**	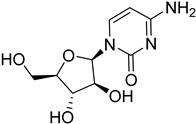	Nucleoside transporter 1 NT1	[34]
**45**	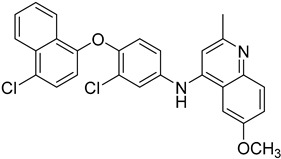	NADH dehydrogenase 2	[105]
**46**	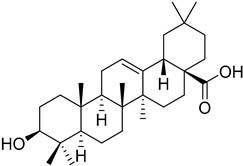	CYP51	[106]
**47**	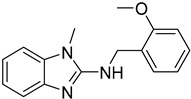	*L*-arginase	[104]
**48**	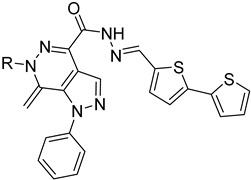	Trypanothione reductase TR1	[117]
**49**	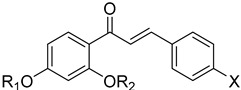	Trypanothione reductase TR1	[118]
**50**	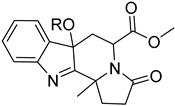	Trypanothione reductase TR1	[119]
**51**	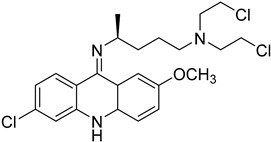	Trypanothione reductase TR1	[107]
**52**	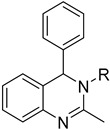	Trypanothione reductase TR1	[107]
**53**	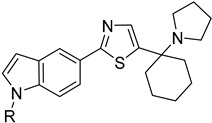	Trypanothione reductase TR1	[107]
**54**	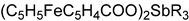	Trypanothione reductase TR1	[108]
**55**	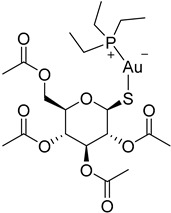	Trypanothione reductase TR1	[107]
**56**	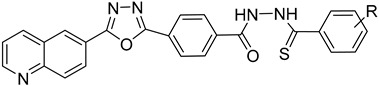	Pteridine reductase PTR1	[110]
**57**	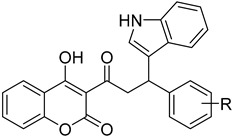	Pteridine reductase PTR1	[112]
**58**	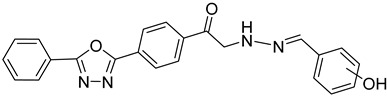	Pteridine reductase PTR1	[111]
**59**	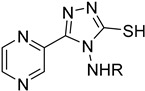	Pteridine reductase PTR1	[113]
**60**	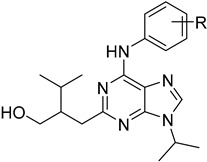	CRK3	[114]
**61**	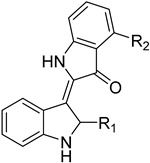	CRK3	[114]
**62**	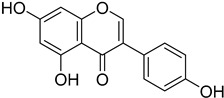	MAPK3	[115]
**63**	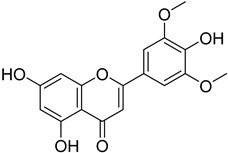	MAPK3	[115]
